# Vancomycin-Resistant Enterococci, Point Barrow, Alaska, USA

**DOI:** 10.3201/eid1505.081219

**Published:** 2009-05

**Authors:** Mirva Drobni, Jonas Bonnedahl, Jorge Hernandez, Paul Haemig, Björn Olsen

**Affiliations:** Uppsala University, Uppsala, Sweden (M. Drobni, J. Bonnedahl, J. Hernandez, B. Olsen); University of Kalmar, Kalmar, Sweden (P. Haemig)

**Keywords:** Antimicrobial resistance, bacteria, vancomycin-resistant enterococci, environment, wild birds, Point Barrow, Alaska, letter

**To the Editor:** An increasing number of bacterial infections are now difficult or impossible to treat (*1*) because of the misuse of antimicrobial drugs and the epidemic spread of bacterial resistance to these drugs (*2*). The most alarming reports are of methicillin-resistant *Staphylococcus aureus*, extended-spectrum β-lactamase producers, and vancomycin-resistant enterococci (VRE). Although knowledge about dissemination mechanisms is poor, the spread of resistance clearly is not restricted to hospitals but occurs also in the community and in the natural environment (*3**,**4*). Since the 1990s, the epidemiology in the United States has shifted so that most VRE are *Enterococcus faecium*. Recent studies indicate clonal spread of the *E. faecium* CC17 lineage in clinical isolates, exhibiting high-level ampicillin and fluoroquinolone resistance and harboring an enterococcal surface protein–coding *esp* gene (*5**,**6*).

During a polar research expedition to the Beringia region in 2005, we collected fecal samples from birds at sites with no or low human population. The aim was to investigate the current status of resistance dissemination into remote areas of the world. The study site in Alaska was located on the tundra halfway between the city of Barrow and Point Barrow, the northernmost point of the United States (71°23′20″N, 156°28′45″W). Fecal samples from glauocus gulls (*Larus hyperboreus*) were enriched (18 h at 37°C) in brain–heart infusion broth (Becton Dickinson, Franklin Lakes, NJ, USA) supplemented with aztreonam and vancomycin (10 mg/L and 4 mg/L, respectively; ICN Biomedicals Inc., Aurora, OH, USA), followed by spreading on chromID VRE plates (bioMérieux, Marcy l’Etoile, France) and incubated for 48 h at 37°C. Typical colonies were isolated and species identified by biochemical testing, including the Phoenix Automated Microbiology System (Becton Dickinson). MIC was determined for vancomycin, teicoplanin, ampicillin, and ciprofloxacin by using Etest strips (AB Biodisk, Solna, Sweden), and the presence of *vanA*, *vanB*, and *esp* genes was established by PCR with previously described primers (*7**,**8*) (esp primers esp11 and esp12).

Cultures showed 2 isolates of *E. faecium*; MICs for vancomycin and teicoplanin were >256 and 96 µg/mL, respectively, for both isolates. Genotyping determined that they harbored *vanA*. Isolates exhibited high-level ampicillin and ciprofloxacin resistance; MICs were >256 and >32 µg mL, respectively for both isolates. They also harbored the *esp* gene. Isolates came from 2 of 33 sampled glaucous gulls, a species confined to the Arctic regions, that have limited southbound migration during the nonbreeding season.

Clinical isolates of VRE were first found in the late 1980s. In the United States, vancomycin was widely used in human medicine, and outbreaks occurred in hospitals rather than in the community; the opposite was, and is, true in Europe. Because of massive use of glycopeptide antimicrobial drugs, i.e., avoparcin, as growth promoters in domestic animal production until the mid-1990s, VRE can be found in hospitals and the community (*9*).

Our findings show that bacteria resistant to antimicrobial drugs, or resistance genes, already have spread to one of the most remote areas of North America, Point Barrow, Alaska. This spread suggests that few (if any) places on earth may be protected against the spread of such resistance, and the dispersal mechanisms are far more efficient than previously thought. Our data also place the isolates as part of the clinically spread clonal *E. faecium* CC17 lineage, characterized by high-level ampicillin and quinolone resistance and harboring the *esp* gene, thus strongly supporting a human origin. Possible dispersal mechanisms to remote areas include stepwise horizontal transfer between migratory and nonmigratory bird species and anthropogenic transport.

The increasing evolution and spread of antimicrobial drug–resistant bacteria and resistance genes seriously threaten public health and could escalate to catastrophic proportions (*1*). Bacteria and drug resistance are easily transferred between humans and animals and consequently between the environment and clinical settings. Much remains to be learned about the effect of human-associated changes of natural ecosystems on the total effect of resistance. Therefore, our finding of VRE at Point Barrow is important to recognize. Decisive action is needed to establish efficient monitoring programs that include not only surveillance and control of clinical bacterial resistance but also environmental levels of resistance.
